# Green Synthesis and Potential Antibacterial Applications of Bioactive Silver Nanoparticles: A Review

**DOI:** 10.3390/polym14040742

**Published:** 2022-02-15

**Authors:** Md. Amdadul Huq, Md. Ashrafudoulla, M. Mizanur Rahman, Sri Renukadevi Balusamy, Shahina Akter

**Affiliations:** 1 Department of Food and Nutrition, College of Biotechnology and Natural Resource, Chung-Ang University, Anseong 17546, Korea; 2 Department of Food Science and Technology, Chung-Ang University, Anseong 17546, Korea; ashrafmiu584@gmail.com; 3 Department of Biotechnology and Genetic Engineering, Faculty of Biological Science, Islamic University, Kushtia 7003, Bangladesh; rahmanmm@btge.iu.ac.bd; 4 Department of Food Science and Biotechnology, Sejong University, Gwangjin-gu, Seoul 05006, Korea; 5 Department of Food Science and Biotechnology, Gachon University, Seongnam 461701, Korea

**Keywords:** green synthesis, silver nanoparticles, antibacterial application, antibacterial mechanisms

## Abstract

Green synthesis of silver nanoparticles (AgNPs) using biological resources is the most facile, economical, rapid, and environmentally friendly method that mitigates the drawbacks of chemical and physical methods. Various biological resources such as plants and their different parts, bacteria, fungi, algae, etc. could be utilized for the green synthesis of bioactive AgNPs. In recent years, several green approaches for non-toxic, rapid, and facile synthesis of AgNPs using biological resources have been reported. Plant extract contains various biomolecules, including flavonoids, terpenoids, alkaloids, phenolic compounds, and vitamins that act as reducing and capping agents during the biosynthesis process. Similarly, microorganisms produce different primary and secondary metabolites that play a crucial role as reducing and capping agents during synthesis. Biosynthesized AgNPs have gained significant attention from the researchers because of their potential applications in different fields of biomedical science. The widest application of AgNPs is their bactericidal activity. Due to the emergence of multidrug-resistant microorganisms, researchers are exploring the therapeutic abilities of AgNPs as potential antibacterial agents. Already, various reports have suggested that biosynthesized AgNPs have exhibited significant antibacterial action against numerous human pathogens. Because of their small size and large surface area, AgNPs have the ability to easily penetrate bacterial cell walls, damage cell membranes, produce reactive oxygen species, and interfere with DNA replication as well as protein synthesis, and result in cell death. This paper provides an overview of the green, facile, and rapid synthesis of AgNPs using biological resources and antibacterial use of biosynthesized AgNPs, highlighting their antibacterial mechanisms.

## 1. Introduction

Nanotechnology is an emerging field of research, with numerous applications in science and technology, especially in the development of different nanomaterials and nanoparticles. Nanoparticles (NPs) are small particles of size from 1 nm to 100 nm and have gained significant interest from scientists due to their multiple applications in diverse fields of science such as biomedicine, agriculture, pharmaceutics, textile, food technology, catalysis, sensors, mechanics, electronics, and optics [1,2]. There are different varieties of nanoparticles, including silver, gold, zinc, cadmium sulfide, copper, iron, titanium dioxide, etc., with unique characteristics [2,3,4,5]. Among different nanoparticles, silver nanoparticles (AgNPs) have been one of the most popular subjects of study in recent decades due to their wide scope of application in different branches of biomedical science as antibacterial, antifungal, antioxidant, anti-cancer, anti-inflammatory, drug delivery, wound dressings, biosensors, and biocatalysis, etc. [6,7,8,9,10,11,12]. Some recent studies have shown the strong antimicrobial, antioxidant, and anti-cancer activities of green synthesized AgNPs [6,7,8]. The biosynthesized AgNPs were also effectively used to degrade various toxic chemicals [9]. Moreover, green synthesized AgNPs have many other applications in different branches of biotechnology such as water filtration, sanitization, food preservation, production of cosmetics, nano-insecticides, and nanopesticides, etc. [10,11,13]. Green synthesized AgNPs have been reported as potential antibacterial agents against various Gram-positive and Gram-negative pathogenic bacteria, including *Salmonella epidermidis*, *Salmonella Typhimurium*, *Pseudomonas aeruginosa*, *Staphylococcus aureus*, *Streptococcus pyogens*, *Escherichia coli*, *Bacillus subtilis*, *Vibrio parahaemolyticus*, *Streptococcus pneumoniae*, *Enterobacter hormaechei*, *Salmonella paratyphi*, *Klebsiella pneumoniae*, *Aeromonas hydrophila*, *Pseudomonas fluorescens*, *Flavobacterium branchiophilum*, *Enterobacter aerogenes*, *Shigella flexneri*, *Xanthomonas axonopodis*, *Salmonella enterica*, etc. [2,3,6,11,12,13].

**Figure 1:**
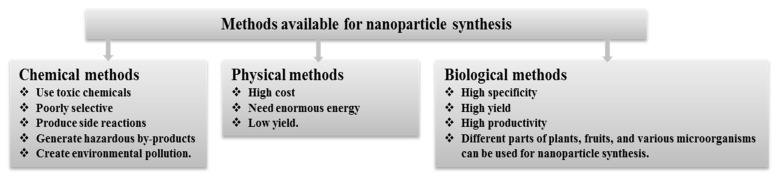
Different methods of nanoparticle synthesis.

Various physical and chemical methods such as physiochemical [14], electrochemical [15], photochemical [16], chemical reduction [17], and microwave irradiation [18] are commonly used for the synthesis of these nanoparticles. The main drawbacks of these methods are that they are expensive and hazardous because of the usage of toxic ingredients, costly, demand labor-intensive equipment and the generation of hazardous byproducts [2,5,19]. Due to the various drawbacks of physicochemical methods, researchers are currently focusing more on biological approaches for eco-friendly, non-toxic, inexpensive, and facile synthesis of nanoparticles (Figure 1). Green synthesis is an efficient process that uses natural compounds as reducing, capping, and stabilizing agents instead of expensive toxic chemicals. Various biological resources such as plants and their different parts (roots, leaves and fruit, etc.), bacteria, fungi, algae, etc. could be utilized for the green synthesis of bioactive nanoparticles [20,21,22,23]. Recently, green synthesis of AgNPs using plant extracts or microbes and their antimicrobial activity were widely investigated.

Multidrug-resistant microorganisms are a serious threat to public health worldwide as different life-threatening infectious diseases are caused by these pathogens. There is a continuous increase in the number of multidrug-resistant bacterial strains due to mutation, pollution, changing environmental conditions and excessive use of drugs. To overcome this problem, scientists are trying to develop new drugs for the treatment of such microbial infections. Green synthesized AgNPs have been found to be effective for controlling these multidrug-resistant bacterial strains. This review provides an overview of green synthesis of AgNPs using different biological resources, various parameters essential for stable, easy and high yields, antibacterial applications and mechanisms of biosynthesized AgNPs as well as describing the prospect for their future development and potential antibacterial applications.

## 2. Green Synthesis of AgNPs

**Figure 2:**
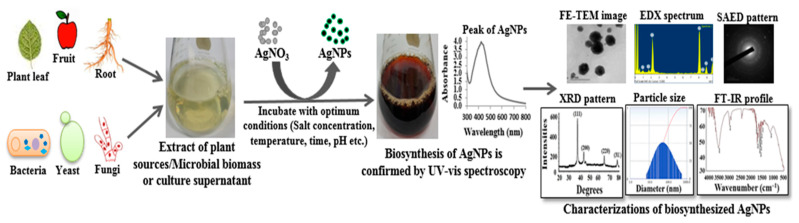
Schematic illustration of green synthesis and characterizations of AgNPs.

Green synthesis of AgNPs using different biological agents such as plants, bacteria, fungi, algae and yeast is an economical, facile, and eco-friendly approach without generating any toxic byproducts. In recent years, both microbes and plants were extensively investigated for the green synthesis of AgNPs. Figure 2 illustrates the various steps of green synthesis of AgNPs using plants and microbes.

## 3. Plant Mediated Synthesis of AgNPs

Plant-mediated synthesis of AgNPs is a widely adopted technique due to the availability of various plants and their easy and safe utilization. Different parts of the plant including fruits, roots, flowers, leaves, peels, etc., have been successfully utilized for the green synthesis of bioactive AgNPs (Table 1). Plant extracts contain numerous bioactive compounds such as alkaloids, flavonoids, terpenoids, tannins, saccharides, phenols, vitamins, as well as various enzymes, amino acids, and proteins [21,24,25]. Due to the presence of these active biomolecules in plant extracts, synthesis of bioactive AgNPs using plants is more stable and easier. In the last few years, many studies have been conducted for the green synthesis of bioactive AgNPs using different parts of plants such as fruits, seeds, roots, flowers, stems, leaves, peels, etc. For instance, the leaf extract of *Clerodendrum viscosum* was used for facile, rapid, and eco-friendly synthesis of bioactive AgNPs [26]. They also investigated the antimicrobial efficacy of biosynthesized AgNPs against various pathogenic bacteria. Pawar and Patil [27] synthesized AgNPs using tuber extract of *Eulophia herbacea*. Fruit extract of *Amomum villosum* was used by Soshnikova et al. [28] for the facile synthesis of AgNPs. The seeds and roots of *Durio zibethinus* and *Rheum palmatum*, respectively, were used for green synthesis of AgNPs [29,30]. Peel extracts of different vegetables such as *Lagenaria siceraria*, *Luffa cylindrica*, *Solanum lycopersicum*, *Solanum melongena* and *Cucumis sativus* were investigated for synthesis of bioactive AgNPs [31]. Synthesis time, size and shape of synthesized AgNPs and their bioactivity varies greatly depending on the plant or part of the plant which was used for synthesis. For example, AgNPs of 10 to 30 nm in size were synthesized using root extract of *Panax ginseng* by two hours’ reaction [32]. On the other hand, AgNPs of 5 to 15 nm were synthesized using leaf extract of *Panax ginseng* within 45 mins of reaction [33]. According to Adeyemi et al. [34], the leaf extract of *Spondias mombin* produced rod- or triangular-shaped AgNPs. However, the plant extract of *Prunus africana*, and *Camellia sinensis* produced spherical-shaped AgNPs [35]. Various parameters such as the extract salt ratio, incubation time, incubation temperature, pH, etc. also greatly affected the easy, rapid, high, and stable synthesis of AgNPs using plant extracts [3,6]. The probable mechanism of plant-mediated synthesis of AgNPs is the chemistry of reduction and oxidation. It has been proposed that the plant extract contains vitamins, amino acids, proteins, enzymes, organic acid, flavonoids, terpenoids, alkaloids, polyphenols, and polysaccharides, which have significant roles for the reduction of silver salts as well as serve as capping and stabilizing agents [21,24,25]. 

## 4. Microbe Mediated Synthesis of AgNPs

In the last few years, the potential of green synthesis of AgNPs using microorganisms has been realized (Table 2). Microorganisms have been shown to be excellent biological agents for the facile, cost effective, and ecofriendly synthesis of AgNPs, avoiding toxic and expensive chemicals and the high energy demands required for physiochemical approaches. Various microorganisms such as bacteria, yeast, fungi, and algae are often favored for the green synthesis of AgNP because of their rapid growth, simpler cultivation and ease of handling. There are two methods for the green synthesis of AgNP using microorganisms, such as the extracellular and intracellular methods [12,24]. Microorganisms synthesize various extracellular and intracellular biomolecules, including amino acid, enzymes, proteins, sugar molecules, organic materials, and many other primary and secondary metabolites [12,24]. The exact mechanism of biosynthesis of AgNP using microorganisms is still not fully known. The widely accepted mechanism of microbe-mediated synthesis of AgNPs is the chemistry of reduction and oxidation, similar to plant-mediated synthesis. First, the metal ions are reduced to NPs with the presence of microbial enzymes including reductase enzyme. Then, various extracellular and intracellular biomolecules of microorganisms serve as the capping and stabilizing agents [2,24]. Huq and Akter [106] have reported the extracellular synthesis of AgNPs from *Massilia* sp. MAHUQ-52. The interaction of 1 mM AgNO_3_ with the bacterial culture supernatant at 30 °C temperature yielded nanoparticles within 48 h of reaction. The size of synthesized AgNPs from FE-TEM analysis was found to range between 15 and 55 nm. 

Singh et al. [108] have demonstrated an extracellular synthesis of AgNPs using the culture supernatant of a bacterial strain *Cedecea* sp. within 48 h of reaction and found spherical-shaped nanoparticles of 10–40 nm in size. Mondal et al. [117] have also reported the rapid synthesis of AgNPs (within 180 min) using the culture supernatants of *Citrobacter* spp. MS5. Another report showed that AgNPs were synthesized through bioreduction of AgNO_3_ by the culture supernatant of *Penicillium chrysogenum* [111]. *Sphingobium* sp. MAH-11 and *Pseudoduganella eburnea* MAHUQ-39 have the ability to produce AgNPs with higher antibacterial activities against pathogenic microbes [2,6]. Eltarahony et al. [107] have reported the intracellular synthesis of AgNPs (within 5 min) using *Streptomyces* strains. Hamida et al. [115] have also reported intracellular synthesis of AgNPs using *Cyanobacteria Desertifilum* sp. They found spherical-shaped nanoparticles of a small size, in the range of 4.5–26 nm. Various fungi and algae were also used for facile, rapid, and ecofriendly synthesis of AgNPs. For instance, the culture supernatant of *Aspergillus terreus* was used to produce AgNPs with a size of 60–100 nm [110]. Raza et al. [112] have reported the intracellular synthesis of AgNPs using a fungus strain *Aspergillus fumigatus* KIBGE-IB33.

## 5. Critical Parameters for Rapid, Facile, and Stable Synthesis of AgNPs

**Figure 3:**
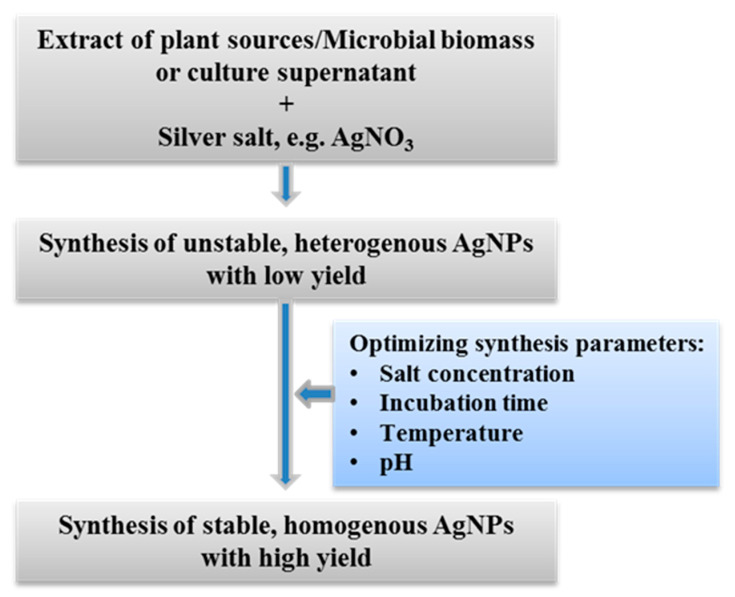
Optimization of parameters for stable, monodispersed, rapid and high-yield of AgNPs.

Several factors play a key role for rapid, stable, and mass production of AgNPs such as the concentration of plant extracts and metal salts, incubation time, temperature, pH, etc. (Figure 3). The shape and size of synthesized nanoparticles also depend on these factors. Extracts of the medicinal plant *Potentilla fulgens* was used by Mittal et al. [72] for the green synthesis of AgNPs and they found that the various physico-chemical parameters including concentrations of plant extract and metal ions, incubation time and temperature, and the pH of the reaction time greatly affected the rate of synthesis as well as their shape, size, and yield. They used different concentrations of plant extract (1 to 200 mg in 50 mL water) and found that 4 mg extract in 50 mL water was able to produce the highest concentration of AgNPs. They also used different concentrations of AgNO_3_ from 0.5 to 5 mM and revealed that the yield of AgNPs increased with the increase of AgNO_3_ concentration from 0.5 to 1 mM, beyond which, there was again a fall in the absorbance. They found that 45°C is the best temperature for maximum yield and concluded that at a higher temperature, the rate of synthesis of smaller size nanoparticles increased. The synthesis was also influenced by the pH of reaction mixture. They revealed that at an alkaline pH, smaller size nanoparticles were formed, whereas at an acidic pH, larger size nanoparticles were observed. Moreover, incubation time had a great effect on the synthesis process as well as the particle size distribution [72]. Nayak et al. [69] have reported the effect of temperature, pH, and incubation time for the green synthesis of AgNPs using bark extracts of *A. indica* and *F. benghalensis* and concluded that 80°C temperature, a pH of 10 and 30 min incubation are the optimum conditions for rapid and stable synthesis. Similarly, Hamouda et al. [3] have shown the effect of plant extracts and AgNO_3_ concentrations for biosynthesis of AgNPs using an aqueous extract of *Oscillatoria limnetica* and reported that concentrations of the aqueous extract of *Oscillatoria limnetica* and AgNO_3_ affected the characteristics of synthesized AgNPs through controlling its size and shape. As with plant-mediated synthesis, microbe-mediated synthesis is also significantly influenced by these parameters. According to Huq [6], extracellular synthesis of AgNPs using culture supernatant of *Pseudoduganella eburnea* MAHUQ-39 was affected by temperature and metal salt (AgNO_3_) concentration. It was found that 30 °C temperature, 1 mM AgNO_3_ (final concentration) and 24 h incubation time are the best conditions for the rapid and stable synthesis of AgNPs using *P. eburnea*. Many other recent studies also showed the effect of concentration of plant extract and metal salt, incubation time, temperature, and pH for the rapid and stable synthesis of homogenous AgNPs with a high yield using both plants and microbes [25,26,48,108].

## 6. Characterization of Green Synthesized AgNPs

Characterization of AgNPs is an important step of green synthesis to check their morphology, size, shape, purity, surface chemistry, etc. Several instruments have been utilized for characterizations of green synthesized AgNPs such as UV-visible spectrophotometry, X-ray diffraction (XRD), Scanning electron microscope (SEM), Transmission electron microscope (TEM), Fourier Transform Infrared Spectroscopy (FTIR), Dynamic light scattering (DLS), and Zeta potential analyzer, etc. Synthesis of AgNPs is initially observed by the naked eye due to the change of color. Generally, the dark brown color of the reaction mixture indicates the synthesis of AgNPs. Then, the formation of AgNPs is confirmed by UV-visible spectrophotometry. Synthesized AgNPs showed a strong peak at around 400–470 nm in UV-visible spectrophotometry. The absorption spectra depended on the morphology, size and shape of biosynthesized of AgNPs [12,140]. SEM and TEM are the powerful tools to characterize the nanoparticles. Both SEM and TEM are used to observe the morphology, shape, size, and the degree of particle aggregation and purity of synthesized nanoparticles [21,108,114]. XRD is an analytical technique which has been utilized to evaluate the structural features of nanoparticles such as the degree of crystallinity, particle sizes, etc. [20]. Dynamic light scattering (DLS) is used to investigate the hydrodynamic size and polydispersity index of synthesized nanoparticles. Measurement of Zeta potential is very important to check the stability of AgNPs in aqueous suspensions. AgNPs with a Zeta potential less than −25 mV or greater than +25 mV typically have high stability [108,141].

**Figure 4:**
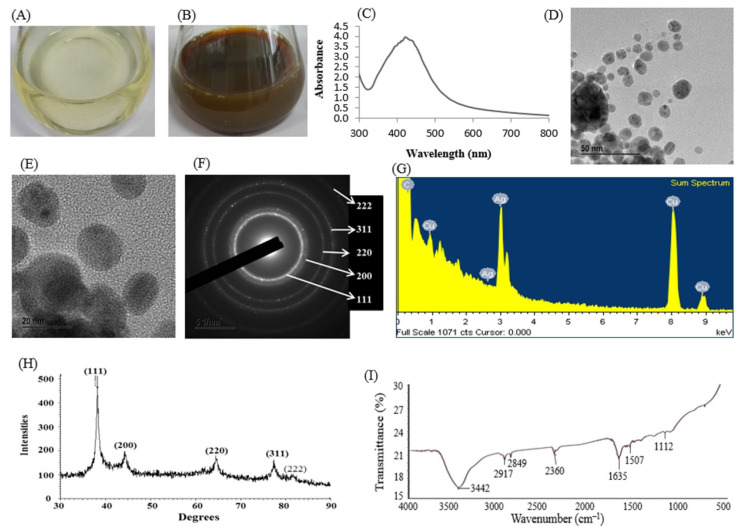
R2A broth with AgNO_3_ as control (**A**); biosynthesized AgNPs (**B**); UV–vis spectra (**C**); FE-TEM images (**D**,**E**); SAED pattern (**F**); EDX spectrum (**G**); X-ray diffraction pattern (**H**); and FT-IR spectra of biosynthesized AgNPs (**I**). This figure has been reprinted with permission from Ref. [2], copyright 2020, Informa UK Limited.

FTIR spectroscopy is a very important tool to investigate the biomolecules responsible for the capping and stabilizing of nanoparticles [2]. Biosynthesis of AgNPs using culture supernatant of *Sphingobium* sp. MAH-11 and their characterization by UV–vis, TEM, XRD, DLS, and FTIR has been reported by Akter and Huq [2]. Synthesis of AgNPs was initially observed by changing of color into dark brown and finally the synthesis was confirmed on the basis of the appearance of a sharp peak at 423 nm in the UV–vis region of the spectrum. The TEM analysis revealed the spherical shape and the size was 7–22 nm. The SAED pattern revealed sharp rings which indicated the crystalline nature of synthesized AgNPs. The XRD pattern also showed the crystalline structure of AgNPs. The FTIR spectrum showed that various biomolecules acted as reducing agents as well as capping and stabilizing agents during the synthesis process (Figure 4), [2]. Sukweenadhi et al. [25] have reported the green synthesis of AgNPs from leaf extract of *Plantago major* and the synthesized AgNPs were characterized by UV–vis, TEM, SEM, XRD, DLS and FTIR. 

## 7. Antibacterial Application of Green Synthesized AgNPs

At the present time, nanoparticles have gained lots of attention by reason of the continuous improvement in treatment of bacterial infections and diseases, as well as inefficient treatment [142]. Among many applied nanoparticles, AgNPs have shown significant application in the reduction of pathogenic microbes and also in the treatment of microbial infections. Due to the rapid increase of antibiotic resistance in this period, this has revived the attention of the researchers investigating the therapeutic abilities of AgNPs systems as potential antimicrobial agents [142]. The published articles proposed the antibacterial activities of AgNPs, and explored them as a promising strategy which could be used as effective growth inhibitors in various microorganisms, antimicrobial control systems and for developing silver-coated medicinal devices, and silver-based dressings, such as nanogels, nanolotions, etc. [12,143,144]. The smaller particle size and greater surface volume of AgNPs holds an extensive contact area with the microbes. These features of AgNPs strongly increases their biological and chemical properties, which greatly helps them to show as robust bactericidal material [145]. This study also showed that AgNPs inhibited the growth of *E. coli* ATCC-15224 on both liquid as well as solid growth media. AgNPs with the concentration of 60 µg/mL have shown a complete cytoxicity to the *E. coli* bacterial strain, and the lower concentration of 60 µg/mL inhibited bacterial cell growth and multiplication [145].

Recently, AgNPs were synthesized using different plants and their various parts as well as bacteria, and the AgNPs produced were tested against various pathogenic microbes including multidrug-resistant bacteria (Table 1 and Table 2). Huq and Akter [106] reported bacterial-mediated synthesis of AgNPs and their antibacterial activity against pathogenic strains of *K. pneumoniae* and *S.* Enteritidis. The synthesized AgNPs showed a 17.6 and a 16.8 mm zone of inhibition (ZOI) against *K. pneumoniae* and *S.* Enteritidis, respectively, whereas some commercial antibiotics such as erythromycin, penicillin, vancomycin, oleandomycin, novobiocin, and lincomycin were resistant or displayed very weak activity against these pathogens. The minimum inhibitory concentration (MIC)/minimum bactericidal concentration (MBC) values of synthesized AgNPs against *K. pneumoniae* and *S. Enteritidis* were 12.5/50.0 and 25.0/50.0 μg/mL, respectively. These MIC/MBC values were well below other antimicrobial agents including zinc oxide and gold nanoparticles against *K. pneumoniae* and *S.* Enteritidis. Another study reported *Arthrobacter bangladeshi* mediated green synthesis of AgNPs and investigated their antibacterial activity against pathogenic strains of *S. typhimurium* and *Y. enterocolitica*. The green synthesized AgNPs showed a 18.3 and a 20.4 mm ZOI against *S. typhimurium* and *Y. enterocolitica*, respectively. The MIC/ MBC values of synthesized AgNPs against *S. typhimurium* and *Y. enterocolitica* were 6.2/12.5 and 3.1/12.5 μg/mL, respectively. These MIC/MBC values were significantly lower than some other antimicrobial agents against *S. typhimurium* and *Y. enterocolitica* [109].

Ahmed et al. [36] reported the green synthesis of AgNPs using dried leaf extract of Tasmanian flax-lily and evaluated their antibacterial activity against several microbes including *S. aureus*, *S. epidermidis*, *P. aeruginosa* and *C. albicans*. *Chlorophytum borivilianum* callus extract was utilized for the green synthesis of AgNPs and the synthesized nanoparticle was used to investigate the antimicrobial activity towards the human pathogens such as *B. subtilis*, *S. aureus*, *P. aeruginosa* and *E. coli*. This result revealed that the synthesized AgNPs showed strong inhibitory activity against tested pathogens [44]. *Plantago major*, *Prunus africana* and *Camellia sinensis* were also reported to synthesize small-size AgNPs and evaluated against *S. aureus*, *E. coli*, *P. aeruginosa* and *K. pneumoniae* [25,35]. It has been reported that smaller size NPs showed higher antibacterial activities due to the larger surface area [146]. It was reported that AgNPs has shown remarkable antibacterial efficacy against antibiotic-resistant human pathogenic strains *S. aureus*, *E. coli*, and *P. aeruginosa* [2]. Hasnain et al. [45] reported on the purple heart plant leaves extract -mediated synthesis of AgNPs and evaluated their antibacterial activity against *E. coli*, and *S. aureus*. They found that the purple heart plant leaves extract-mediated synthesized AgNPs showed significantly strong antibacterial activity against both *E. coli*, and *S. aureus* compared to the purple heart plant leaves extract. Another report also proposed the excellent antimicrobial activity of biosynthesized AgNPs against various Gram-negative and Gram-positive pathogenic microorganisms which showed the way to use it as a potential application of antibacterial agent against multidrug-resistant bacteria [126].

Recently a few studies have stated that the conjugation of AgNPs with bactericidal agents may reduce the toxic effect towards the mammalian cells whilst increasing the bactericidal activity. This conjugation helps to increase the amount of antibacterial agent in the specific bacterial site and thus the therapeutic activity of the antibiotic agents could be enhanced against the bacterial infection [147,148]. It was also reported that AgNPs can be applied on a clinical platform against human pathogenic strains *C. albicans*, *S. enterica*, *E. coli*, and *V. parahemolyticus* [134,135]. It was demonstrated that green synthesis AgNPs has shown antimicrobial activity against multidrug-resistant pathogenic microbes. They also mentioned that it was ecofriendly, safe, facile, effective, and economical, which could be applied in both medical and non-medical sectors, especially as an antimicrobial agent to control drug-resistant pathogens [20]. The biosynthesized AgNPs presented great antimicrobial effect against multidrug-resistant pathogens such as *S. aureus* and *P. aeruginosa*. The MBCs to inhibit *S. aureus* and *P. aeruginosa* were 200 and 50 µg/mL, respectively [6]. In another study this author proposed that the AgNPs synthesized by strain MAHUQ-40 showed significant antibacterial activity against *V. parahaemolyticus* and *S. Typhimurium* with MICs 3.12 and 6.25 µg/mL, respectively [114], whereas some commercial antibiotics such as penicillin G, erythromycin, oleandomycin, lincomycin, and vancomycin were resistant or displayed very weak activity against these pathogens. Another study investigated antimicrobial activity against both Gram-positive *B. cereus* and Gram-negative bacteria *P. aeruginosa*. The bacterial-mediated synthesized AgNPs inhibited the growth of pathogenic strains *B. cereus* and *P. aeruginosa* through developing a clear zone of inhibition [12]. Due to this killing ability, AgNPs are recognized for their remarkable antibacterial activity. Moreover, the modification in AgNPs surface developed the interactions of the constituents and this surface modification of AgNPs through chemical functionalization has gained much consideration which could be useful in numerous areas such as medical, engineering, and biological uses [149,150].

## 8. Antibacterial Mechanisms of AgNPs

The most important thing about nanoparticles is their mechanism of action and this mechanism mostly depends upon the size, pH, and ionic strength of the medium, and also on the type of capping agent. However, the complete antibacterial mechanism of AgNPs is still not fully known and has not been completely explained. According to the previous studies, it could be considered that AgNPs may frequently release the silver ions (Ag+), which might be considered as one of the mechanisms behind the bactericidal activity of AgNPs [142,151]. It has been demonstrated that the Ag+ ion forms complexes with the nucleic acids and interacts with the nucleosides of nucleic acids to show antibacterial activities. Nanoparticles altered the membrane permeability as evident from the release of sugars, proteins, and nuclear material through the damaged membrane [152]. The electrostatic attractions as well as an affinity towards the sulfur proteins enhanced the adhesion of Ag+ ion to the cytoplasm and cell membrane and lead to the disruptions of bacterial casings with enhancing the permeability of bacterial cell membrane [153]. 

**Figure 5:**
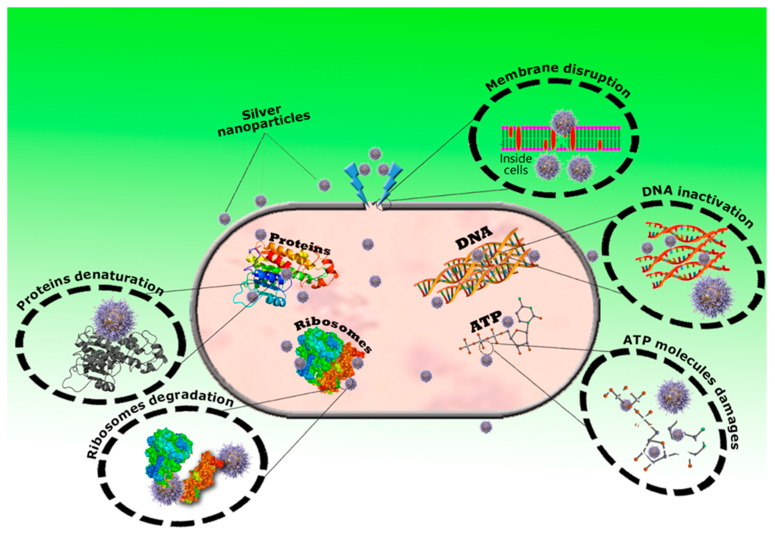
Possible antibacterial mechanisms of AgNPs. Disruption of cell wall and cell membrane, damage of ATP molecules due to the production of reactive oxygen species, DNA inactivation, protein denaturation and ribosome degradation.

The production of reactive oxygen species (ROS) is increased due to the production of free Ag+ ions by the cells, which may interrupt adenosine triphosphate (ATP) release [154]. This ROS may play an important role to disrupt the cellular membrane and the alteration in the deoxyribonucleic acid, which could cause different issues related to DNA, including DNA replication and cell propagation. On the other hand, free Ag+ ions may efficiently interfere the protein synthesis by denaturing cytoplasmic ribosomal components [155]. The release percentage of Ag+ ions can inhibit the growth of bacteria because the nanoscale size of AgNPs has the ability to penetrate the bacterial cell wall as well as denaturation of the cell membranes [156]. Due to denaturation of the cell membrane the intracellular and extracellular components of bacterial cell membrane may be ruptured which also causes cell lysis [157]. The antibacterial mechanisms of the AgNPs are mainly influenced by their dissolution profile in the reaction media and dissolution efficacy also depend on the synthesis and processing parameters [158]. Although the exact antibacterial mechanism of AgNPs has not been entirely clarified, different antibacterial actions of AgNPs have been proposed in Figure 5. 

**Figure 6:**
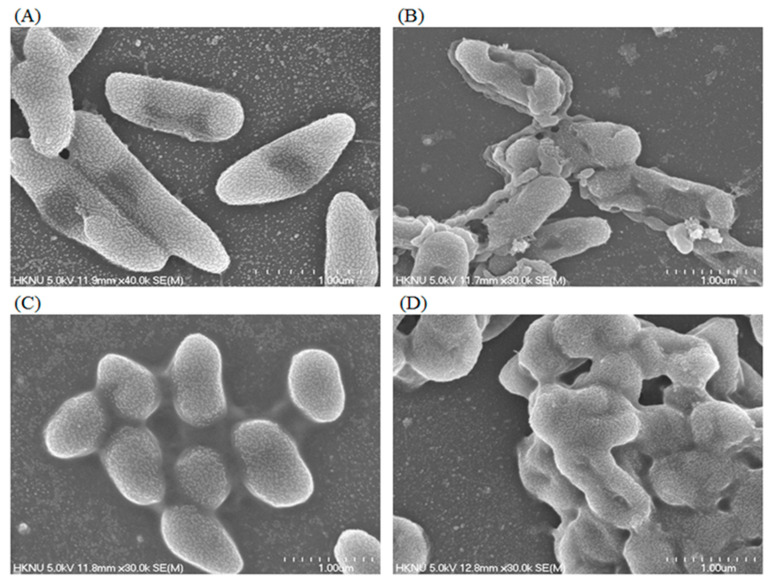
FE–SEM images of normal *P. aeruginosa* cells (**A**); 1 × MBC AgNPs treated *P. aeruginosa* cells (**B**); normal *S. aureus* cells (**C**); 1 × MBC AgNPs treated *S. aureus* cells (**D**). This figure has been reprinted with permission from Ref. [6], copyright 2020, MDPI.

The researchers proposed that the biosynthesized AgNPs may affect the bacterial cell morphology and penetrate the cell membrane by damaging the of cell wall of *E. coli* and *S. aureus*, which may decrease the reproduction of cell and ultimately lead to cell death. The FE-SEM images proved the strong antibacterial mechanism of AgNPs against pathogenic bacteria and promoted the application of AgNPs as an antimicrobial agent [2]. Another study demonstrated that synthesized AgNPs changed the structural function of bacterial cells like *S. aureus* and *P. aeruginosa*. These mechanical activities of the proposed AgNPs create a promising hope to recognize it as an effective antimicrobial agent for various therapeutic applications against *S. aureus* and *P. aeruginosa* infections [6]. It was stated that AgNPs show the efficacy to alter the cell morphology as well as damage the cell membrane of tested pathogens (Figure 6). The main mechanism of AgNPs is that these nanoparticles strongly attach to the bacterial cell membrane surface and disturb its proper function, because of the enhancement of DNA damage [126]. The AgNPs also have the capability to penetrate the cell membrane and when it penetrates the cell membrane it potentially disrupts the cellular components by reacting with the sulphur-mediated proteins and phosphorus-mediated complexes like deoxyribonucleic acid [159]. Scanning electron microscopy (SEM) and TEM studies demonstrated that AgNPs shown the ability to adhere and interact with *E. coli* and penetrate into the bacterial cells. This adhesion and interaction ability increases the antibacterial activity of AgNPs, which are attributed with total surface area of nanoparticles [145]. Thus, the ecofriendly synthesis of AgNPs could be useful in various applications in both pharmaceutical and non- pharmaceutical sectors to eradicate drug-resistant pathogens [20]. 

AgNPs have changed and damaged *V. parahaemolyticus* and *S. typhimurium* membrane integrity, which reduced the metabolic activity and normal cell function caused bacterial cells’ death [114]. The field emission scanning electron microscopy analysis demonstrated that AgNPs were responsible for damaging the cell wall and altering the cell morphology of treated Gram-positive and Gram-negative pathogenic bacteria, leading to the death of cells [12]. The literature demonstrated that AgNPs trigger the inhibition of protein synthesis as well as cell wall synthesis, which provides strong evidence about the protein disruption of the outer cellular membrane and increasing ATP leakage, resulting in cell death [160]. 

Apart from these, the size and shape of the AgNPs increase the release of Ag+ ions owing to their greater surface area which influence potential activity against bacterial disease. The dissolution rate of AgNPs also interferes with its antimicrobial level. If the dissolution rate is high, then the potential activity could be increased [161]. It is generally proposed that AgNPs smaller than 10 nm may directly penetrate cell membranes, enter into the bacterial cells, and initiate cell lysis [162]. Therefore, the finding may provide a meaningful statement about AgNPs to use as an alternative antibacterial agent to protect pathogenic bacteria as well as to treat bacterial infectious diseases. 

## 9. Conclusions and Future Prospects

Green synthesis of AgNPs is preferred due to its eco-friendly nature. The utilization of various parts of plant, bacteria, fungi, algae is an efficient, facile and environmentally friendly way to synthesize AgNPs. Plant extracts contain different biomolecules such as amino acids, proteins, enzymes, terpene, alkaloids, flavonoids, phenols, tannins, vitamins, etc., which act as reducing, capping, and stabilizing agents. Similarly, microorganisms synthesize various extracellular and intracellular biomolecules such as enzymes, amino acid, proteins, and many other primary and secondary metabolites that act as reducing agents as well as capping and stabilizing agents during the synthesis process. Biosynthesized AgNPs have great bactericidal potential against various Gram-positive and Gram-negative bacteria. In this review, green synthesis of AgNPs using plants and microbes has been comprehensively reviewed. The antibacterial applications and mechanisms of the biosynthesized AgNPs against pathogenic microbes have also been highlighted. Although the rapid, facile and eco-friendly synthetic methods using plants and microbes have shown great potential in AgNPs, the exact mechanism of synthesis and the mode of antimicrobial action are still not fully understood. Hence, several points might be considered for the future synthesis of AgNPs from plants or microbes. First, the selection of plant or microbes for easy, rapid and eco-friendly synthesis. For plant selection, researchers should consider the availability of plants and their extraction process. Plants should be available and the extraction process should be simple for facile and mass production of AgNPs. Similarly, researchers should focus on non-pathogenic and rapid growth microbes for safe and easy handling during the synthesis process. In this case, probiotic microbes could be the great synthetic agent. Second, investigation of the biomolecules present in plant extracts or in microbial biomass or culture supernatant. It is believed that different biomolecules present in plant extracts or in microbial culture supernatant are mainly responsible for the synthesis and stabilization of AgNPs. The role of various enzymes for biosynthesis needs to be studied in detail. Additionally, these biomolecules are also responsible to enhance the antibacterial efficacy of synthesized AgNPs. Therefore, it is important to investigate the biomolecules present in the plant extract or in microbial culture supernatant for successful synthesis of AgNPs. Third, optimization of parameters for rapid, stable and mass production of AgNPs. Several studies reported that various parameters such as concentration of the plant extract and AgNO_3_, incubation time and temperature, pH of reaction, etc. have great effect on synthesis process. Hence, mass production on an industrial scale can be achieved by optimizing these reaction conditions. Fourth, investigation of the antibacterial mechanisms. Most of the studies reported the efficacy of AgNPs in the screening level without investigating the exact mechanisms. It is very important to find out the mode of action of AgNPs against pathogens. Fifth, investigation of cytotoxic effect of biosynthesized AgNPs on human cells. Some studies reported that AgNPs have cytotoxic effects on human cells. Hence, it is essential to investigate the potential toxicity of biosynthesized AgNPs on healthy human cells to ensure their safe use for human and the environment. 

## Figures and Tables

**Table 1:** Green synthesis of AgNPs using plants and their antibacterial applications.

Plants	Used Parts	Size (nm)	Shape	Optimum Synthesis Parameters	Target Pathogens	References
*Plantago major*	Leaf extract	10−20	Spherical	1 mM, 70 °C, 60 min	*S. aureus*, *E. coli*, *P. aeruginosa*	[25]
*Prunus africana*, *Camellia sinensis*	Plant extract	10−19	Spherical	0.5 mM, 25 °C, 24 h	*E. coli*, *K. pneumoniae*	[35]
Tasmanian flax-lily	Dried leaves extract	Av. 70	Spherical	0.1 mM, 60 °C, 25 min	*S. aureus*, *S. epidermidis*, *P. aeruginosa*, *C. albican*	[36]
*Carduus crispus*	Plant extract	33–131	NA	1 mM, room temperature, 24 h	*E. coli*, *M. luteus*	[37]
*Anastatica hierochuntica*, *Artemisia absinthium*	Plant and seed extracts	Av. 114, 125.5	Spherical	1 mM, room temperature, 48 h	*P. aeruginosa*, *E. coli*, *S. aureus*, *C. albicans*	[38]
*Lantana trifolia*	Leaf extract	5–70	Spherical	1.5 M, 35 °C, 2 h	*S. aureus*, *C. albicans*, *E. coli*, *P. aeruginosa*, *B. subtilis*	[39]
*Blumea eriantha*	Plant extract	10–60	Spherical	1%, ambient temperature, 24 h	*S.aureus*, *B. subtilis*, *B. cereus*, *E. coli*	[40]
*Cucumis prophetarum*	Leaf extract	30−50	Polymorphic	1 mM, 80 °C, 3 h	*S. typhi*, *S. aureus*	[41]
*Clerodendrum viscosum*	Leaf extract	36−74	Spherical	1 mM, 60 °C, 60 min	*E. coli*, *P. aeruginosa*, *B. subtilis*, *S. aureus*	[26]
Grape	Proanthocyanidin from seed	100−120	Aggregated	Ambient temperature, 2–3 h	*S. aureus*, *P. aeruginosa*, *E. coli*	[42]
*Spondias mombin*	Leaf extract		Rod or triangular	1 mM, room temperature	*S. aureus*, *P. aeruginosa*, *E. coli*	[34]
*Eulophia herbacea*	Tuber extract	Av. 11.7	NA	1 mM, room temperature, 5 h	*E. coli*, *S. aureus*, *P. aruginosa*, *B. subtilis*	[27]
*Torreya nucifera*	Leaf extract	10–125	Spherical	1 M, 20 °C, 24 h	*S. typhimurium*	[43]
*Chlorophytum borivilianum*	Callus extracts	35–168	Spherical	1 mM, room temperature, 5 h	*B. subtilis*, *S. aureus*, *P. aeruginosa*, *E. coli*	[44]
Purple heart plant	Leaves extract	Av. 104.6	NA	50 mM, 65 °C	*E. coli*, *S. aureus*	[45]
*Phoenix dactylifera*	Root hair extract	21–41	Spherical	0.1 mM, 50 °C, 48 h	*C. albicans*, *E. coli*	[46]
*Taraxacum officinale*	Leaf extract	5–30	Spherical	1 mM, room temperature, 15 min	*X. axonopodis*, *P. syringae*	[47]
Chicory	Seed exudates	≤25	Spherical	5 mM, 30 °C	*P. aeruginosa*, *K. pneumoniae*, *A. baumannii*, *F. solani*	[48]
*Punica granatum*	Peel extract	20–40	Spherical	0.1 mM, room temperature, 72 h	*E. coli*, *S. epidermidis*, *P. aeruginosa*, *S. typhi*, *P. vulgaris*, *S. aureus*, *K. pneumonia*	[49]
*Durio Zibethinus*	Seed extract	20–75	Spherical, rod	1.5 mM, in sunlight, 30 min	*E. coli*, *B. subtilis*, *S. typhimurium*, *S. typhi*	[29]
Market vegetable	Vegetable waste extract	10–90	Spherical	1 mM, 37 °C, 5 h	*Klebsiella* sp., *Staphylococcus* sp.	[50]
*Rheum palmatum*	Root extract	44–113	Hexagonal, spherical	2 mM, room temperature, 24 h	*S. aureus*, *P. aeruginosa*	[30]
*Angelica pubescens*	Root extract	20–50	Quasi-spherical	5 mM, 80 °C, 50 min	*S. aureus*, *P. aeruginosa*, *E. coli*, *S. enterica*	[51]
*Protium serratum*	Leaf extract	Av. 74.5	Spherical	1 mM, 25 °C, 4 h	*P. aeruginosa*, *E. coli*, *B. subtilis*	[52]
*Amomum villosum*	Dried fruit extract	5–15	Spherical	1 mM, room temperature, 3 s	*S. aureus*, *E. coli*	[28]
*Glycyrrhiza uralensis*	Root extract	5–15	Spherical	1 mM, 80 °C, 40 min	*E. coli*, *S. aureus*, *P. aeruginosa*, *S. enterica*	[53]
*Ficus palmata*	Leaf extract	28–33	Spherical	2 mM, room temperature, 6 h	*S. pneumonia*, *E. coli*, *P. aeruginosa*, *K. pneumonia*, *P. vulgaris*	[54]
*Euphorbia antiquorum*	Latex extract	10–50	Spherical	1 mM, room temperature, 24 h	*K. Pneumoniae*, *P. mirabilis*, *V. cholerae*, *E. faecalis*	[55]
*Ocimum Sanctum*	Leaf extract	Av. 14.6	Spherical	2 mM, 35 °C, 4 h	*E. coli*	[56]
*Moringa stenopetala*	Leaf extract	Av. 11.4	NA	1 mM, 60 °C, 15 min	*S. aureus*, *E. coli*	[57]
*Euphrasia officinalis*	Leaf extract	Av. 40.3	Quasi-spherical	1 mM, 65 °C, 19 min	*P. aeruginosa*, *E. coli*, *S. aureus*, *V. parahaemolyticus.*	[58]
*Siberian ginseng*	Dried stem	Av. 14.6	Spherical	1 mM, 80 °C, 1.5 h	*S. aureus*, *B. anthracis*, *V. parahaemolyticus.* *E. coli*	[59]
*Borago officinalis*	Leaf extract	30–80	Spherical, hexagonal, irregular	1 mM, 65 °C, 68 s	*P. aeruginosa*, *E. coli*, *V. parahaemolyticus*, *S. aureus*	[60]
*Cocoa pod*	Husk extract	4–32	Spherical	1 mM, 30°C, few minutes	*E. coli*, *K. pneumoniae*, *S. pyogenes*, *S. aureus*, *P. aeruginosa*	[61]
*Lagenaria siceraria*, *Luffa cylindrica*, *Solanum lycopersicum*, *Solanum melongena*, *Cucumis sativus*	Vegetable peel extract	up to 20	Spherical	2 mM, 80 °C, 10 min	*E. coli*, *K. pneumoniae*	[31]
*Azadirachta indica*	Leaf extract	Av. 34	Spherical	1 mM, room temperature, 24 h	*S. aureus*, *E. coli*	[62]
*Pedalium murex*	Leaf extract	10–50	Spherical	10 mM, 20 min	*B. subtilis*, *S. aureus*, *E. coli*, *M. flavus*, *P. aeruginosa*, *B. pumilus*, *K. pheumoniae*	[63]
*Cassia fistula*	Leaf extract	40–50	Spherical	1 mM, room temperature, overnight	*B. subtilis*, *S. aureus*, *C. kruseii*, *T. mentagrophytes*	[64]
*Psidium guajava*	Leaf extract	10–90	Spherical	1 mM, 30 °C, 10 min	*P. aeruginosa*	[65]
*Coffea arabica*	Seed extract	10–150	Spherical, ellipsoidal	20 mM, room temperature, 2 h	*E. coli*, *S. aureus*	[66]
*Styrax benzoin*	Benzoin gum extract	12–38	Spherical	1 mM, 60 °C, 5 h	*E. coli*, *P. aeruginosa*, *S. aureus*, *C. tropicalis*	[21]
*Cardiospermum halicacabum*	Leaf extract	Av. 23	Cubic	1 mM, room temperature, 16 h	*P. vulgaris*, *P. aeruginosa*, *S. aureus*, *B. subtilis*, *S. paratyphi*, *A. solani*, *F. oxysporum*	[67]
*Atrocarpus altilis*	Leaf extract	20–50	Spherical	1 mM, 25 °C, 24 h	*S. aureus*, *P. aeruginosa*, *E. coli*, *A. vesicolor*	[68]
*Ficus benghalensis*, *Azadirachta indica*	Bark extracts	Av. 60	Spherical	1 M, 80 °C, 30 min	*E. coli*, *P. aeruginosa*, *V. cholera*, *B. subtilis*	[69]
*Thevetia peruviana*	Leaf extract	Av. 18.1	Spherical	1 mM, 30 °C, 4 h	*E. coli*, *K. pneumonia*, *P. aeruginosa*, *S. aureus*, *B. subtilis*, *S. typhi*	[70]
*Capparis spinosa*	Leaf extract	5–30	Spherical	10 mM, room temperature, 15 min	*E. coli*, *S. typhimurium*, *S. aureus*, *B. cereus*	[71]
*Potentilla fulgens*	Root extract	10–15	Spherical	1 mM, 35 °C, 18 h	*E. coli*, *B. subtilis*	[72]
*Petroselinum crispum*	Leaf extract	30–32	Spherical	10 mM, room temperature, 24 h	*K. pneumoniae*, *E. coli*, *S. aureus*	[73]
*Eucalyptus* *globulus*	Leaf extract	5–25	Spherical, oval	1 mM, 37 °C, 60 min	*P. aeruginosa*, *E. coli*, *S. aureus*	[74]
Banana plant	Banana peel extract	23.7	Spherical	1.75 mM, 30 °C, 72 h	*E. coli*, *P. aeruginosa*, *B. subtilis*, *S. aureus*	[75]
*Zingiber officinale*	Rhizome	1.4–5.7	Spherical	1 mM, room temperature, 1 h	*S. aureus*, *E. coli*	[76]
*Erythrina indica*	Root extract	20–118	Spherical	1 mM, room temperature, overnight	*S. aureus*, *M. luteus*, *E. coli*, *B. subtilis*, *S. typhi*, *S. paratyphi*	[77]
*Prosopis farcta*	Plant extract	Av. 10.8	Spherical	1 mM, room temperature, 1 h	*S. aureus*, *B. subtilis*, *E. coli*, *P. aeruginosa*	[78]
*Cassia roxburghii*	Aqueous extract	10–30	Spherical	1 mM, room temperature, overnight	*B. subtilis*, *S. aureus*, *M. luteus*, *P. aeruginosa*, *E. coli*, *E. aerogenes*	[79]
*Garcinia mangostana*	Fruit extract	30–50	Various	1 mM, 80 °C, 15 min	*E. coli*, *P. aeruginosa*, *S. aureus*	[80]
*Panax ginseng*	Root extract	10–30	Spherical	1 mM, 80 °C, 2 h	*B. anthracis*, *E. coli*, *V. parahaemolyticus*, *S. aureus*, *B. cereus*	[32]
*Panax ginseng*	Leaf extract	5–15	Spherical	1 mM, 80 °C, 45 min	*E. coli*, *S. enterica*, *V. parahaemolyticus*, *S. aureus*, *B. anthracis*, *B. cereus*	[33]
*Clitoria ternatea*, *Solanum nigrum*	Leaf extract	20–28	Spherical	100 mM, room temperature, 60 min	*B. subtilis*, *S. aureus*, *S. pyogenes*, *E. coli*, *P. aeruginosa*, *K. aerogenes*	[81]
*Mukia maderaspatana*	Leaf extract	Av. 158	Spherical	1 mM, room temperature, 15–20 min	*B. subtilis*, *K. pneumoniae*, *S. aureus*, *S. typhi*	[82]
*Terminalia arjuna*	Plant extract	8–16	Spherical	1 mM, room temperature, 15 min	*S. aureus*, *E. coli*	[83]
*Eclipta alba*	Leaf extract	310–400	Cubic	1 mM, 32 °C, 24 h	*E. coli*, *S. aureus*, *P. aeruginosa*	[84]
*Alternanthera dentata*	Leaf extract	50–100	Spherical	1 mM, 60 °C, 45 min	*E. coli*, *P. aeruginosa*, *K. pneumonia*, *E. faecalis*	[85]
*Dalbergia spinosa*	Leaf extract	Av. 18	Spherical	100 mM, room temperature, 30 min	*B. subtilis*, *P. aeruginosa*, *S. aureus*, *E. coli*,	[86]
*Pulicaria glutinosa*	Plant extract	40–60	Spherical	1 mM, 90 °C, 2 h	*E. coli*, *P. aeruginosa*, *S. aureus*, *M. luteus*	[87]
*Phyllanthus amarus*	Aqueous extract	15.7–29.9	Spherical	1 mM, 70 °C, 20 min	*P. aeruginosa*	[88]
*Withania somnifera*	Leaf powder	5–30	Spherical	1 mM, room temperature, 12 h	*S. aureus*, *E. coli*	[89]
*Acorous calamus*	Rhizome extract	Av. 31.8	Spherical	1 mM, room temperature, 12 h	*B. subtilis*, *B. cereus*, *S. aureus*	[90]
*Cocos nucifera*	Plant extract	Av. 22	Spherical	0.9 mM, 36 °C, 24 h	*K. pneumoniae*, *B. subtilis*, *P. aeruginosa*, *S. paratyphi*	[91]
*Boerhaavia diffusa*	Plant extract	Av. 25	Spherical	100 mM, 24 h	*A. hydrophila*, *P. fluorescens*, *F. branchiophilum*	[92]
*Azadirachta indica*	Leaf extract	4.7–18.9	Spherical	0.1 N, room temperature, 2 h	*B. subtilis*, *S. typhimorium*	[93]
*Coriandrum sativum*	Seed extract	9.9–12.6	Spherical	0.1 N, room temperature, 2 h	*B. subtilis*	[94]
*Hibiscus cannabinus*	Leaf extract	7 –25	Spherical	5 mM, 30°C, 40 min	*E. coli*, *P. mirabilis*, *S. flexneri.*	[95]
*Ocimum tenuiflorum*	Leaf extract	25–40	NA	1 mM, room temperature, 10 min	*E. coli*, *Corney bacterium*, *B. substilus*	[96]
*Tribulus terrestris*	Fruit bodies	16–28	Spherical	1 mM, room temperature, 36 h	*S. pyogens*, *P. aeruginosa*, *E. coli*, *B. subtilis*, *S. aureus*	[97]
*Lantana camara*	Fruit extract	12.5–13.0	Spherical	1 mM, room temperature, 1 h	*M. luteus*, *B. subtilis*, *S. aureus*, *V. cholerae*, *K. pneumoniae*, *S. typhi*	[98]
*Morinda citrifolia*	Leaf extract	10–60	Spherical	1 mM, 90 °C, 60 min	*E. coli*, *P. aeroginosa*, *K. pneumoniae*, *E. aerogenes*, *B. cereus*, *Enterococci* sp.	[99]
*Terminalia chebula*	Plant extract	less than 100	Pentagons, spherical, triangular	2 mM, room temperature, 15–20 min	*S. aureus*, *E. coli*	[100]
*Solanum xanthocarpum*	Berry extract	4–18	Spherical	1 mM, 45 °C, 25 min	*H. pylori*	[101]
*Dioscorea bulbifera*	Tuber extract	8–20	Nanorods, triangles	0.7 mM, 50 °C, 5 h	*E. coli*, *P. aeruginosa*, *S. typhi*, *B. subtilis*	[102]
*Garcinia mangostana*	Leaf extract	Av. 35	Spherical	1 mM, 75 °C, 60 min	*E. coli*, *S. aureus*	[103]
*Cymbopogan citratus*	Leaf extract	Av. 32	Spherical	1 mM, 37 °C, 24 h	*E.coli*, *S. aureus*, *P. mirabilis*, *S. typhi*, *K. pnuemoniae*	[104]
*Sesuvium portulacastrum* L.	Callus and leaf extracts	5–20	Spherical	1 mM, room temperature, 24 h	*P. aeruginosa*, *S. aureus*, *L. monocytogenes*, *M. luteu*, *K. pneumoniae*, *A. alternata*, *P. italicum*, *F. equisetii*, *C. albicans*	[105]

Av., average; NA, not available; s, second; min, minute; h, hour.

**Table 2:** Green synthesis of AgNPs using microorganisms and their antibacterial applications.

Microorganisms	Method	Size (nm)	Shape	Optimum Synthesis Parameters	Target Pathogens	References
*Massilia* sp. MAHUQ-52	Extracellular	15–55	Spherical	1 mM, 30 °C, 48 h	*K. pneumoniae*, *S. Enteritidis*	[106]
*Streptomyces* strains	Intracellular	1.17–13.3	Spherical	5 mM, 30 °C, 120 h	*B. cereus*, *E. faecalis*, *S. aureus*, *E. coli*, *S. typhi*, *P. aeruginosa*, *K. pneumoniae*, *P. vulgaris*	[107]
*Cedecea* sp.	Extracellular	10–40	Spherical	2 mM, 37 °C, 48 h	*E. coli*, *P. aeruginosa*, *S. epidermis*, *S. aureus*	[108]
*Arthrobacter* *bangladeshi*	Extracellular	12–50	Spherical	1 mM, 30 °C, 24 h	*S. typhimurium*, *Y. enterocolitica*	[109]
*Aspergillus terreus*	Extracellular	60–100	Spherical	100 mM, 27 °C, 48 h	*K. pneumoniae*, *S. aureus*, *S. typhi*, *P. aeruginosa*, *E. coli*, *S. epidermidis*, *E. faecalis*, *P. mirabilis*, *B. subtilis*	[110]
*Penicillium* *chrysogenum*	Extracellular	18–60	Spherical	1 mM, 28 °C, 24 h	*C. albicans*, *C. krusei*, *C. tropicalis*, *C. parapsilosis*, *C. glabrata*	[111]
*Paenarthrobacter nicotinovorans*	Extracellular	13–27	Spherical	1 mM, 30 °C, 24 h	*B. cereus*, *P. aeruginosa*	[12]
*Aspergillus fumigatus*	Intracellular	<100	Spherical	3.5 mM, 25 °C, 72 h	11 different pathogenic bacteria	[112]
*Paenibacillus* sp.	Extracellular	17.4–52.8	Spherical	0.1 mM, room temperature, 120 h	*S. aureus*, *E. faecalis*, *S. pneumoniae*, *E. coli*	[113]
*Lysinibacillus xylanilyticus*	Extracellular	8–30	Spherical	1 mM, 30 °C, 48 h	*V. parahaemolyticus*, *S. Typhimurium*	[114]
*Cyanobacteria Desertifilum* sp.	Intracellular	4.5–26	Spherical	1 mM, room temperature, 24 h	*B. cereus*, *P. aeruginosa*, *B. cercus*, *B. subtilis*, *S. flexneri*, *S enterica*	[115]
*Chlorella ellipsoidea*	Intracellular	Av. 220	Spherical, cubic, rod, triangular	1 mM, room temperature, 24 h	*S. aureus*, *E. coli*, *K. pneumoniae*, *P. aeruginosa*	[116]
*Citrobacter* spp. MS5	Extracellular	5–15	Spherical	1 mM, 40 °C, 180 min	*E. hormaechei*, *K. pneumoniae*	[117]
*Sphingobium* sp. MAH-11	Extracellular	7–22	Spherical	1 mM, 30 °C, 48 h	*E. coli*, *S. aureus*	[2]
*Padina* sp.	Intracellular	25–60	Spherical	10 mM, 60 °C, 48 h	*S. aureus*, *B. subtilis*, *P. aeruginosa*, *S. typhi*, *E. coli*	[118]
*Chaetoceros* sp., *Skeletonema* sp., *Thalassiosira* sp.	Biomass	149–239	Rectangular, square, regular	room temperature, 48 h	*E. coli*, *B. subtilis*, *S. pneumonia*, *Aeromonas* sp., *S. aureus*	[119]
*Penicillium oxalicum*	Extracellular	60–80	Spherical	1 mM, 37 °C, 72 h	*S. aureus*, *S. dysenteriae*, *S. typhi*	[120]
*Lactobacillus plantarum*	Intracellular	Av. 14.0	Spherical	2 mM, 37 °C, 24 h	*S. aureus*, *E. coli*, *S. epidermidis*, *Salmonella* sp.	[121]
*Escherichia coli*	Extracellular	5–50	Spherical	1 mM, 37 °C, 72 h	*B. subtilis*, *S. aureus*, *B. cereus*, *P. aeruginosa*, *K. pneumoniae*, *E. coli*, *S. typhi*, *E. vermicularis*	[122]
*Terrabacter humi*	Extracellular	6–24	Spherical	1 mM, 30 °C, 48 h	*E. coli*, *P. aeruginosa*	[20]
*Bacillus subtilis*	Intracellular	3–20	Spherical	1 mM, 30 °C, 24 h	*S. aureus*, *E. coli*, *S. epidermidis*, *K. pneumoniae*, *C. albicans*	[123]
*Pseudoduganella eburnea* MAHUQ-39	Extracellular	8–24	Spherical	1 mM, 30 °C, 24 h	*S. aureus*, *P. aeruginosa*	[6]
*Oscillatoria* *limnetica*	Extracellular	3.3–17.9	quasi-spherical	10 mM, room temperature, 48 h	*E. coli*, *B. cereus*	[3]
*Acinetobacter baumannii*	Extracellular	37–168	Spherical	1 mM, 37 °C	*E. coli*, *P. aeruginosa*, *K. pneumoniae*	[124]
*Pseudomonas* sp. THG-LS1.4	Extracellular	10–40	Irregular	1 mM, 28 °C, 48 h	*B. cereus*, *S. aureus*, *C. tropicalis*, *V. parahaemolyticus*, *E. coli*, *P. aeruginosa*	[125]
*Novosphingobium* sp. THG-C3	Extracellular	8–25	Spherical	1 mM, 25 °C, 48 h	*S. aureus*, *C. tropicalis*, *P. aeruginosa*, *E. coli*, *V. parahaemolyticus*, *C. albicans*, *S. enterica*, *B. subtilis*, *B. cereus*	[126]
*Sporosarcina koreensis* DC4	Extracellular	30–50	Spherical	1 mM, 25 °C, 48 h	*V. parahaemolyticus*, *E. coli*, *S. enterica*, *B. anthracis*, *B. cereus*, *S. aureus*	[127]
*Bacillus* sp. AZ1	Extracellular	7–31	Spherical	1 mM, 40 °C, 24 h	*S. typhi*, *E. coli*, *S. epidermis*, *S. aureus*	[128]
*Aeromonas* sp. THG-FG1.2	Extracellular	8–16	Spherical	1 mM, 28 °C, 48 h	*S. enterica*, *E. coli*, *P. aeruginosa*, *V. parahaemolyticus*, *B. cereus*, *B. subtilis*, *S. aureus*, *C. albicans*	[129]
*Kinneretia* THG-SQI4	Extracellular	15–20	Spherical	1 mM, 28 °C, 48 h	*C. albicans*, *E. coli*, *C. tropicalis*, *B. cereus*, *B. subtilis*, *S. aureus*, *S. enterica*, *P. aeruginosa*, *V. parahaemolyticus*	[130]
*Bacillus safensis*	Extracellular	5–30	Spherical	1 mM, 30 °C, 2 h	*E. coli*	[131]
*Aspergillus niger*	Intracellular	43–63	Spherical	1 mM, 35 °C, 48 h	*K. planticola*, *E. coli*, *Pseudomonas* sp., *B. subtilis*, *B. cereus*	[132]
*Weissella oryzae*	Extracellular	10–30	Spherical	1 mM, 25 °C, 48 h	*V. parahaemolyticus*, *B. cereus*, *B. anthracis*, *S. aureus*, *E. coli*, *C. albicans*	[133]
*Microbacterium resistens*	Extracellular	10–20	Spherical	1 mM, 30 °C, 48 h	*S. enterica*, *S. aureus*, *B. anthracis*, *B. cereus*, *E. coli*, *C. albicans*	[134]
*Bacillus methylotrophicus*	Extracellular	10–30	Spherical	1 mM, 28 °C, 48 h	*V. parahaemolyticus*, *S. enterica*, *E. coli*, *C. albicans*	[135]
*Pseudomonas deceptionensis*	Extracellular	10– 30	Spherical	1 mM, 25 °C, 48 h	*S. aureus*, *S. enterica*, *V. parahaemolyticus*, *B. anthracis*, *C. albicans*	[136]
*Bhargavaea indica*	Extracellular	30–100	Pentagon, spherical, hexagonal, triangle, nanobar	1 mM, 25 °C, 48 h	*V. parahaemolyticus*, *S. enterica*, *S. aureus*, *B. anthracis*, *B. cereus*, *E. coli*, *C. albicans*	[137]
*Actinomycetes*	Extracellular, Intracellular	65–80	Spherical	1 mM, 37 °C, 72 h	*S. aureus*, *E. coli*, *K. pneumoniae*, *P. vulgaris*, *P. aeruginosa*	[138]
*Bacillus flexus*	Extracellular	12–61	Spherical, triangular	1 mM, room temperature, 8 h	*S. pyogenes*, *B. subtilis*, *P. aeruginosa*, *E. coli*	[139]

## Data Availability

Not applicable.
